# Multi-Probe RFA vs. Single-Probe MWA in an Ex Vivo Bovine Liver Model: Comparison of Volume and Shape of Coagulation Zones

**DOI:** 10.3390/biology12081103

**Published:** 2023-08-08

**Authors:** Gregor Laimer, Michael Bauer, Yannick Scharll, Peter Schullian, Reto Bale

**Affiliations:** Interventional Oncology-Microinvasive Therapy (SIP), Department of Radiology, Medical University Innsbruck, Anichstr. 35, 6020 Innsbruck, Austria; gregor.laimer@i-med.ac.at (G.L.);

**Keywords:** radiofrequency ablation, ablation techniques, liver

## Abstract

**Simple Summary:**

The aim of this study was to compare the volumes and shapes of the coagulation zone (CZ) of a multi-probe RFA system (three RFA electrodes) and a single-probe MWA system from the same vendor in an ex vivo bovine liver model. Radiofrequency ablation and microwave ablation are both minimally invasive treatment options for liver cancer. A total of 48 CZs were obtained in bovine liver specimens in three different ablation system configurations and compared at different ablation times using a fixed ablation protocol. Ablation diameters were measured and ellipticity indices (EIs) and volumes calculated. Volumes and diameters increased with ablation time for all configurations. The single-probe MWA could not reach the volumes of the RFA setups for any of the ablation times evaluated. EI were very similar and almost round for RFA with 20 mm inter-probe distance and single-probe MWA and differed significantly to the three individual, more ovoid ones for the RFA 50 mm configuration. In this ex vivo study, the multi-probe RFA system employing three electrodes achieved significantly larger ablation volumes in both configurations (confluent CZ and three individual CZ) per time as compared with a single-probe MWA system.

**Abstract:**

Objectives: To compare the volumes and shapes of the coagulation zone (CZ) of a multi-probe RFA system (three RFA electrodes) and a single-probe MWA system from the same vendor in an ex vivo bovine liver model. Material & Methods: A total of 48 CZs were obtained in bovine liver specimens with three different ablation system configurations (single-probe MWA vs. multi-probe RFA with 20 mm inter-probe distance [confluent CZ] vs. multi-probe RFA with 50 mm inter-probe distance [three individual CZs]) at 4, 6, 8, and 10 min ablation time using a fixed ablation protocol. Ablation diameters were measured and ellipticity indices (EIs) and volumes calculated. Calculations for all systems/configurations were compared. Results: Volumes and diameters increased with ablation time for all configurations. At 4 and 6 min ablation time volumes obtained with the RFA 50 mm setup, and at 8 and 10 min with the RFA 20 mm setup were the largest at 26.5 ± 4.1 mL, 38.1 ± 5.8 mL, 46.3 ± 4.9 mL, 48.4 ± 7.3 mL, respectively. The single-probe MWA could not reach the volumes of the RFA setups for any of the ablation times evaluated. EI were very similar and almost round for RFA 20 mm and single-probe MWA, and differed significantly to the more ovoid ones for the RFA 50 mm configuration. Conclusions: The multi-probe RFA system employing three electrodes achieved significantly larger ablation volumes in both configurations (confluent CZ and three individual CZs) per time as compared with a single-probe MWA system in this ex vivo bovine liver model.

## 1. Introduction

Radiofrequency ablation (RFA) and microwave ablation (MWA) are minimally invasive, potentially local curative treatment options for primary and secondary liver tumors [1,2,3]. MWA is becoming increasingly popular as it allows for a larger ablation zone per time unit and probe. However, with emerging new technologies and sophisticated tools multiple coaxial needles may be precisely positioned as place holders for simultaneous (by employing up to three RFA electrodes) and sequential thermal ablation [4]. Using this stereotactic RFA (SRFA) technique even very large tumors (>10 cm) can be treated within one session [5]. Therefore, the benefits and drawbacks of these two different approaches have to be reevaluated.

Over the past two decades, numerous ex vivo, in vivo, and clinical studies have evaluated various ablation systems and configurations and their effects on ablation volume. While earlier studies on RFA compared different activation methods [6], power settings [7], and amount and duration of applied energy [8,9], RFA systems [10], or size of electrodes [11], more recent studies also evaluated the influence of tissue properties on the ablated volume. For example, Cassinotto et al. [12] investigated the so-called “oven effect” in a retrospective in-vivo analysis and concluded that the presence of cirrhosis did not affect the ablated volume. In fact, RFA-induced coagulation zones in cirrhotic livers were of comparable size and volume to those in healthy livers.

In the last decade, more studies have been conducted on ablation volumes using MWA due to the increased use of this method. Ruiter et al. [13] have recently published a systematic review on various FDA-approved systems. They compared data of 14 studies and found a significant correlation between ablation volume and applied energy for ex vivo experiments, but not for in-vivo experiments.

However, to the best of our knowledge, currently there is no ex vivo study comparing ablated volumes of multi-probe RFA with single-probe MWA. Thus, the purpose of this study was to evaluate the differences between a multi-probe RFA system with three electrodes in two different configurations and an MWA system from the same vendor in terms of ablated volumes and coagulation zone shape in an ex vivo bovine liver model.

## 2. Materials and Methods

### 2.1. Overview

The experiment was performed with 13 fresh bovine livers (total weight 70 kg) purchased from a local butcher. They were stored refrigerated, not frozen, and allowed to attain room temperature overnight before being used. On the day of the experiment, the bovine livers were placed in a plastic container filled with a 0.9% saline solution prepared fresh daily with 62 L of water and sodium chloride. The temperature of the 0.9% saline solution was monitored during the experiment with a thermometer to detect and counteract possible temperature increases above room temperature. The ablation probes were inserted into the bovine liver specimen through drilling holes in a 20 mm thick acrylic pane. The ablation systems were activated for different periods according to the protocol and corresponding coagulation zones were evaluated on longitudinal and orthogonal cuts of the ablation zones along the probe axis.

### 2.2. Experimental Setup

Two widely used ablation systems (RFA: Cool-tip; 25 cm, 3 cm, 17 G; MWA: Emprint, 15 cm, 3 cm, 13 G) from the same vendor (Medtronic Inc. (Boulder, CO, USA)) were utilized for this experiment. 

All ablations were generated in fresh ex vivo bovine liver specimens (10 × 10 × 10 cm).

For the setup a total of three containers were used:

- Container #1 (57 × 38 × 47 cm) served as a large basin for the 0.9% saline solution. The distance between the tip of the ablation probe and grounding electrode was 20 cm in order to avoid irregular ablation zones.
- Container #2 was used to hold the bovine liver specimen (10 × 10 × 10 cm).
- Container #3 was used to fix the bovine liver specimen.

In order to allow communication of the fluid volumes between the different containers, container #2 had 24 holes with a diameter of 6 mm at the bottom, and multiple large openings on each side. It was positioned on a stainless chrome steel frame (20 × 14.5 × 27 cm). Container #3 had the same size as the bovine liver specimen in order to fix it. Each side of container #3 was also perforated several times.

To achieve circulation in the system, a submersible pump (JBL ProFlow t300, 4 watt, 300 L/h, lift 0.5 m, Out 12/16) was positioned in container #1.

The ablation probes were guided through an acrylic glass pane (thickness 20 mm) fixed with clamps on container #1.

**Figure 1:**
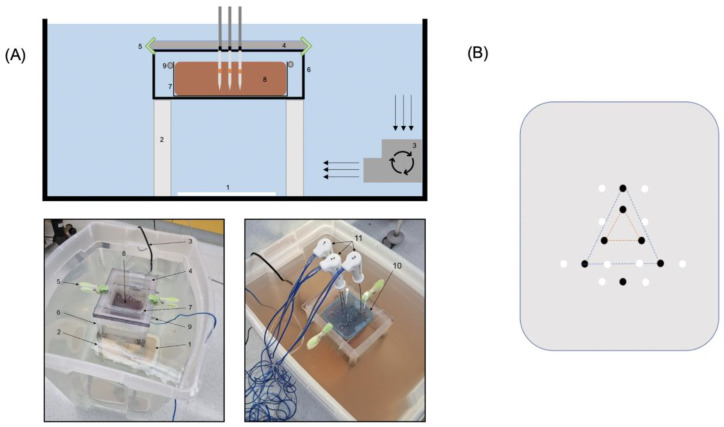
(**A**) Schematic illustration and corresponding images of the experimental setup with RFA 50 mm setup as example: (1) neutral electrode, (2) stainless chrome steel frame, (3) submersible pump, (4) acrylic glass pane, (5) clamps, (6) container #2, (7) container #3, (8) liver specimen, (9) stainless bars, (10) blue lid as positioning aid, (11) RFA electrodes. (**B**) Schematic illustration of pre-drilled acrylic glass pane (20 mm thickness) to guarantee exact positioning of probes. Holes for ablation probes (black) and k-wires (white, at 15 mm distance) with RFA 20 mm setup (orange triangle), RFA 50 mm setup (blue triangle), and a separate hole for single-probe MWA.

The experimental setup is shown in Figure 1A.

### 2.3. Arrangement of Probes

To guarantee an exact and reproducible position and correct angle in the liver specimens, all probes were inserted through a pre-drilled acrylic glass pane with 20 mm thickness.

The RFA electrodes were inserted in a triangular shape at a distance of 20 mm and 50 mm (orange and blue triangle, Figure 1B), thus either creating a confluent coagulation zone (20 mm distance) or three individual coagulation zones (50 mm distance). For the single MWA probe, a separate hole was drilled.

After ablation, two k-wires were inserted at each side of the coagulation zone at a distance of 15 mm from the center of the triangle (confluent coagulation zone) or to the electrodes/probes (individual coagulation zone RFA/MWA) to ensure an accurate and reproducible cutting plane (see Figure 1B).

### 2.4. Ablation Protocol

RFA (Cool-tip; 25 cm, 3 cm, 17 G): three probes were arranged in the form of a triangle with 20 mm and 50 mm distance with settings according to the manufacturer’s instructions (program: “Standard Ablation”; delivery of energy to the tissue via an automatic impedance control algorithm; max. output 200 W). Four runs were performed in each configuration for 4, 6, 8, and 10 min each, respectively.

MWA (Emprint, 15 cm, 3 cm, 13 G): a single probe with power settings according to manufacturer’s instructions (100 W) was used. Four runs were performed for 4, 6, 8, and 10 min, respectively.

### 2.5. Evaluation of the Coagulation Zones

**Figure 2:**
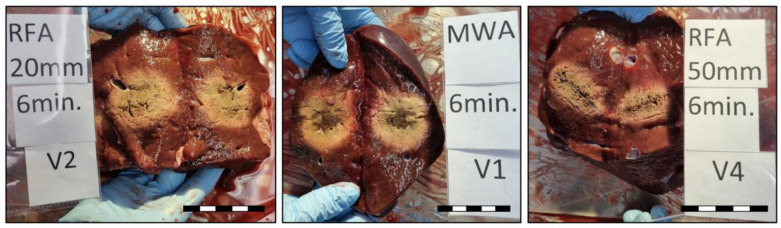
Example of sectioned liver specimen along the axis of the coagulation zones at 6 min for RFA 20 mm setup (confluent CZ), MWA, and RFA 50 mm setup (individual CZ). A ruler of 5 cm in each bottom right corner with correct proportions.

After ablation, the liver specimens were sectioned along the axis of the ablation probe, using the K-wires inserted after ablation as guides (see examples in Figure 2).

**Figure 3:**
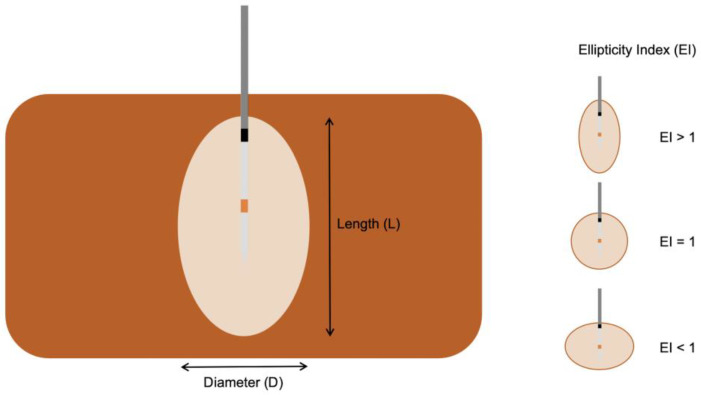
Schematic illustration of coagulation zone evaluation with length, diameter, and ellipticity index.

The coagulation zone dimensions were measured using a Vernier caliper. The diameter (D) was defined as the maximal diameter of the ablation zone measured in the plane perpendicular to the ablation probe. The length (L) was defined as the distance between proximal/distal edges of the ablation zone in the axis of the ablation probe. Ablation volumes in mL (assuming an ellipsoidal shape) were calculated using the formula (4/3 × π × a × b × c). Ellipticity indices (EIs) were calculated using the formula: L/D [14]—see schematic illustration Figure 3.

In the RFA 50 mm setup the length, diameter, and EI of each coagulation zone were evaluated for each coagulation zone individually (*n* = 12) and the three volumes of the individual coagulation zones were summed to a single total volume (*n* = 4) at each time point.

### 2.6. Statistics

All statistical analyses were performed using the SPSS Version 28 (SPSS Inc., Chicago, IL, USA).

The distribution (parametric/non-parametric) of all possible variables was assessed using histograms and verified with the Kolmogorov-Smirnov test. Data are expressed as mean ± SD. Differences between devices/arrangements were assessed using a one-way ANOVA with Bonferroni’s post hoc test or student’s independent *t*-test, as appropriate. A *p*-value of <0.05 was considered statistically significant.

## 3. Results

To achieve the planned 48 coagulation zones, including four in each configuration [MWA, RFA 20 mm, and RFA 50 mm] and for each ablation time [4, 6, 8, and 10 min], a total of 59 coagulation zones were created, of which 11 had to be excluded due to distortion by a larger nearby vessel (MWA: *n* = 2 ; RFA 20 mm: *n* = 4 ; RFA 50 mm: *n* = 5).

**Figure 4:**
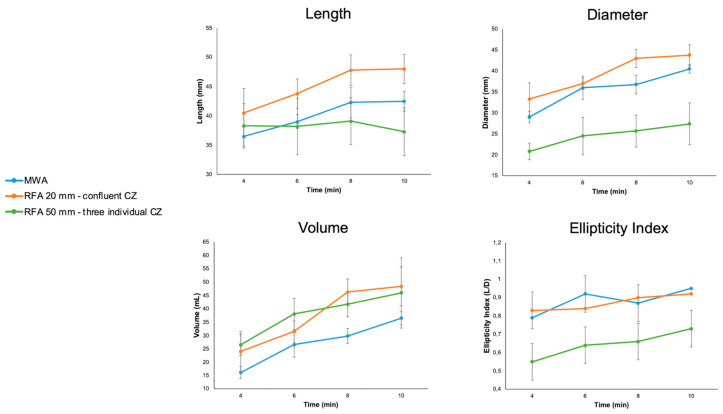
Descriptive of length, diameter, volume, and ellipticity index for each ablation system/configuration and ablation time (4, 6, 8, and 10 min); mean values ± standard deviation.

Overall results with mean values of length, diameter, volume, and ellipticity index for different ablation systems and times are summarized in Table 1 and illustrated in Figure 4.

### 3.1. MWA

#### 3.1.1. Volume

The largest ablated volume using MWA was achieved at 10 min ablation time with 36.5 ± 2.6 mL. Volumes of the coagulation zone differed significantly between ablation times (16.1 ± 2.2 mL [4 min] vs. 26.6 ± 4.7 mL [6 min] vs. 29.8 ± 2.8 mL [8 min] vs. 36.5 ± 2.6 mL [10 min]; *p* < 0.001), increasing from 4 to 6 min by 65.2%, from 6 to 8 min by 12.0%, and from 8 to 10 min by 22.5%. Post hoc analysis revealed significant differences in the ablation volumes between 4 vs. 6 min (*p* = 0.004), 4 vs. 8 min (*p* < 0.001), 4 vs. 10 min (*p* < 0.001), and 6 vs. 10 min (*p* = 0.006)—but not for 6 vs. 8 min and 8 vs. 10 min.

#### 3.1.2. Diameter

The largest diameter with the single-probe MWA system was achieved at 10 min ablation time with 40.5 ± 1.0 mm. Diameters differed significantly between ablation times (29.0 ± 1.4 [4 min] vs. 36.0 ± 2.8 mm [6 min] vs. 36.8 ± 2.2 mm [8 min] vs. 40.5 ± 1.0 [10 min]; *p* < 0.001), increasing from 4 to 6 min by 24.1%, from 6 to 8 min by 2.2%, and from 8 to 10 min by 10.1%. Post hoc analysis revealed significant differences of the ablation volumes between 4 vs. 6 min (*p* = 0.002), 4 vs. 8 min (*p* < 0.001), 4 vs. 10 min (*p* < 0.001), and 6 vs. 10 min (*p* = 0.047)—but not for 6 vs. 8 min and 8 vs. 10 min.

#### 3.1.3. Ellipticity Index

Coagulation zones became significantly more spherical with increasing ablation time (1.3 ± 0.0 [4 min] vs. 1.1 ± 0.0 [6 min] vs. 1.2 ± 0.1 [8 min] vs. 1.0 ± 0.0 [10 min]; *p* = 0.013) and were the roundest at 10 min. Post hoc analysis revealed significant differences between 4 vs. 10 min (*p* = 0.016) only.

### 3.2. RFA 20 mm—Confluent Coagulation Zone

#### 3.2.1. Volume

The largest ablated volume using this setup was achieved at 10 min ablation time with 48.4 ± 7.3 mL. Volumes of the coagulation zone differed significantly between ablation times (24.0 ± 7.6 mL [4 min] vs. 31.5 ± 4.0 mL [6 min] vs. 46.3 ± 4.9 mL [8 min] vs. 48.4 ± 7.3 mL [10 min]; *p* < 0.001), increasing from 4 to 6 min by 31.3%, from 6 to 8 min by 47.0%, and from 8 to 10 min by 4.5%. Post hoc analysis revealed significant differences between 4 vs. 8 min (*p* = 0.002), 4 vs. 10 min (*p* < 0.001), 6 vs. 8 min (*p* = 0.032), and 6 vs. 10 min (*p* = 0.013)—but not for 4 vs. 6 min and 8 vs. 10 min.

#### 3.2.2. Diameter

The largest diameter using this setup was achieved at 10 min ablation time with 48.4 ± 7.3 mm. Diameters differed significantly between ablation times (33.3 ± 3.9 mm [4 min] vs. 37.0 ± 1.4 mm [6 min] vs. 43.0 ± 2.2 mm [8 min] vs. 43.8 ± 2.5 mm [10 min]; *p* < 0.001), increasing from 4 to 6 min by 11.1%, from 6 to 8 min by 16.2%, and from 8 to 10 min by 1.9%. Post hoc analysis revealed significant differences between 4 vs. 8 min (*p* = 0.001), 4 vs. 10 min (*p* < 0.001), 6 vs. 8 min (*p* = 0.048), and 6 vs. 10 min (*p* = 0.023)—but not for 4 vs. 6 min or 8 vs. 10 min.

#### 3.2.3. Ellipticity Index

Coagulation zones were almost identical in elliptic shape and no statistically significant differences were found (1.2 ± 0.1 [4 min] vs. 1.2 ± 0.0 [6 min] vs. 1.1 ± 0.0 [8 min] vs. 1.1 ± 0.0 [10 min]; *p* = 0.145). 

### 3.3. RFA 50 mm—Three Individual Coagulation Zones

#### 3.3.1. Volume

The largest ablated volume (sum of three individual coagulation zones) using this setup was achieved at 10 min ablation time with 46.0 ± 13.2 mL. The sum of volumes of the three coagulation zones differed significantly between ablation times (26.5 ± 4.1 mL [4 min] vs. 38.1 ± 5.8 [6 min] vs. 41.7 ± 4.7 mL [8 min] vs. 46.0 ± 13.2 mL [10 min]; *p* = 0.024), increasing from 4 to 6 min by 43.8%, from 6 to 8 min by 9.4%, and from 8 to 10 min 10.3% (8 to 10 min). Post hoc analysis revealed significant differences only between 4 vs. 10 min (*p* = 0.026). 

#### 3.3.2. Diameter

The largest diameter (single CZ) using this setup was achieved at 10 min ablation time with 27.4 ± 5.0 mm. The diameter of the single coagulation zones in this setup differed significantly between ablation times (20.8 ± 2.0 mm [4 min] vs. 24.5 ± 4.5 mm [6 min] vs. 25.7 ± 3.8 mm [8 min] vs. 27.4 ± 5.0 mm [10 min]; *p* = 0.002), increasing from 4 to 6 min by 17.8%, from 6 to 8 min by 4.9%, and from 8 to 10 min by 6.6% (8 to 10 min). Post hoc analysis revealed significant differences only between 4 vs. 8 min (*p* = 0.027) and 4 vs. 10 min (*p* = 0.001).

#### 3.3.3. Ellipticity Index

Coagulation zones at 10 min were the roundest and became significantly more spherical with increasing ablation time (1.8 ± 0.1 [4 min] vs. 1.6 ± 0.1 [6 min] vs. 1.5 ± 0.1 [8 min] vs. 1.4 ± 0.1 [10 min]; *p* < 0.001). Post-hoc analysis revealed significant differences between 4 vs. 8 min (*p* = 0.018), 4 vs. 10 min (*p* < 0.001)—but not when comparing the remaining ablation times. 

### 3.4. MWA vs. RFA 20 mm vs. RFA 50 mm—Volume

When comparing all volumes regardless of system and ablation time, the largest ablated volume was achieved using the RFA 20 mm (confluent CZ) setup at 10 min with 48.4 ± 7.3 mL.

#### 3.4.1. Volumes at 4 Min

At 4 min ablation time, the largest ablated volume was achieved with the RFA 50 mm setup with 26.5 ± 4.1 mL. One-way ANOVA revealed a significant difference of ablated volumes between systems/setups (16.1 ± 2.2 mL [MWA], 24.0 ± 7.6 mL [RFA 20 mm], 26.5 ± 4.1 mL [RFA 50 mm]; *p* = 0.048). However, in post hoc analysis no significant differences were found.

#### 3.4.2. Volumes at 6 Min

At 6 min ablation time, the largest ablated volume was achieved using the RFA 50 mm setup with 38.1 ± 5.8 mL. One-way ANOVA revealed a significant difference in ablated volumes between systems/setups (26.6 ± 4.7 mL [MWA], 31.5 ± 4.0 mL [RFA 20 mm], 38.1 ± 5.8 mL [RFA 50 mm]; *p* = 0.027). Post hoc analysis revealed significant differences in ablated volumes for MWA vs. RFA 50 mm (*p* = 0.027)—but not for MWA vs. RFA 20 mm and RFA 20 mm vs. RFA 50 mm.

#### 3.4.3. Volumes at 8 Min

At 8 min ablation time, the largest ablated volume was achieved using the RFA 20 mm setup with 46.3 ± 4.9 mL. One-way ANOVA revealed a significant difference in ablated volumes between systems/setups (29.8 ± 2.8 mL [MWA], 46.3 ± 4.9 mL [RFA 20 mm], 41.7 ± 4.7 mL [RFA 50 mm]; *p* = 0.001). Post hoc analysis revealed significant differences in ablated volumes for MWA vs. RFA 20 mm (*p* = 0.001) and MWA vs. RFA 50 mm (*p* = 0.010)—but not for RFA 20 mm vs. RFA 50 mm.

#### 3.4.4. Volumes at 10 Min

At 10 min ablation time, the largest ablated volume was achieved with the RFA 20 mm setup with 48.4 ± 7.3 mL. One-way ANOVA revealed no significant difference in ablated volumes between systems/setups (36.5 ± 2.6 mL [MWA], 48.4 ± 7.3 mL [RFA 20 mm], 46.0 ± 13.2 mL [RFA 50 mm]; *p* = 0.188). 

**Figure 5:**
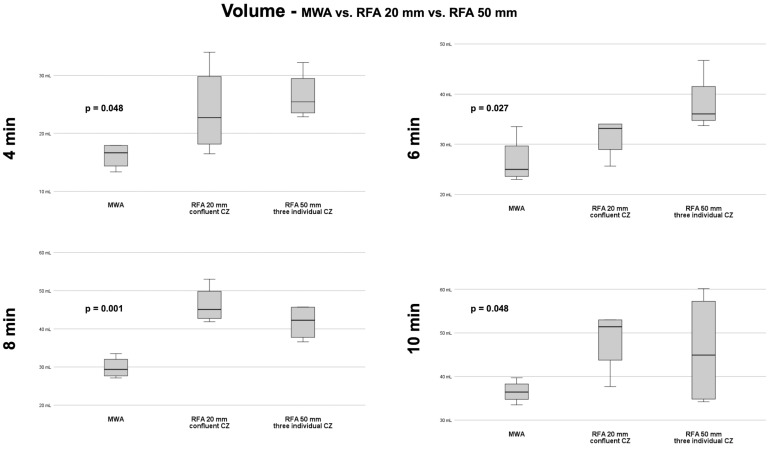
Box plots with comparison of volumes of ablation systems/configurations for each ablation time with corresponding *p*-values of one-way ANOVA.

Comparison of all volumes between systems/setups at each ablation time is depicted in Figure 5.

### 3.5. MWA vs. RFA 20 mm vs. RFA 50 mm—Diameter

Overall, the largest diameter was achieved with the RFA 20 mm setup at 10 min ablation time with 43.8 ± 2.5 mm. Diameters differed significantly between systems/setups for all evaluated ablation times (4 min [*p* < 0.001]; 6 min [*p* < 0.001]; 8 min [*p* < 0.001]; 10 min [*p* < 0.001]). Post hoc analyses revealed significant differences between the RFA 50 mm setup (diameter of a single CZ) compared with the other two systems/setups for all ablation times. Between MWA and the RFA 20 mm setup, diameters differed significantly only at 8 min ablation time (36.8 ± 2.2 vs. 43.0 ± 2.2 [*p* = 0.018] with no differences for any of the other ablation times observed. 

**Figure 6:**
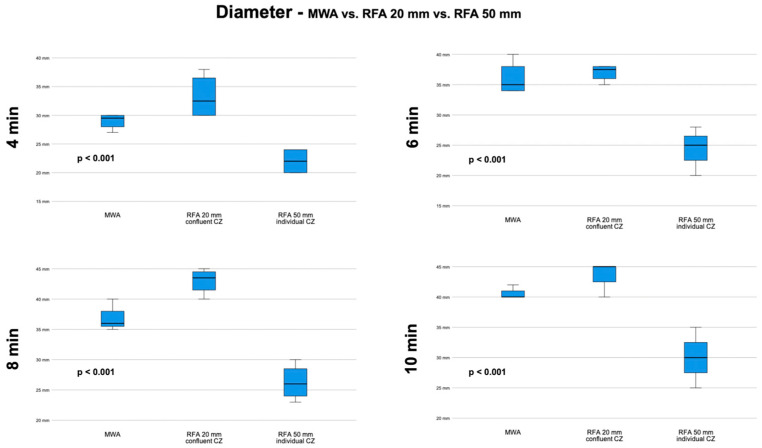
Box plots with comparison of diameters of ablation systems/configurations for each ablation time with corresponding *p*-values of one-way ANOVA.

Diameters at each ablation time with comparison between systems/setups is illustrated in Figure 6.

### 3.6. MWA vs. RFA 20 mm vs. RFA 50 mm—Ellipticity Index

Overall, the roundest coagulation zone was obtained with the MWA system at 10 min ablation time with 1.0 ± 0.0 and the most ovoid coagulation zone was observed with the RFA 50 mm setup at 4 min ablation time with 1.8 ± 0.1.

Ellipticity Index differed significantly between systems/setups for all evaluated ablation times (4 min [*p* < 0.001]; 6 min [*p* < 0.001]; 8 min [*p* = 0.001]; 10 min [*p* = 0.044]). Post-hoc analyses revealed significant differences between MWA and RFA 50 mm for all ablation times (1.3 ± 0.0 vs. 1.8 ± 0.1, *p* < 0.001 [4 min]; 1.8 ± 0.1 vs. 1.6 ± 0.1, *p* < 0.001 [6 min]; 1.2 ± 0.1 vs. 1.5 ± 0.1, *p* < 0.004 [8 min]) except for 10 min (1.0 ± 0.0 vs. 1.4 ± 0.1, *p* = 0.060). Between RFA 20 mm and RFA 50 mm ellipticity index differed significantly only at 8 min ablation time (1.1 ± 0.0 vs. 1.5 ± 0.1, *p* = 0.002), while there were no significant differences in ellipticity index between MWA and RFA 20 mm for any ablation time.

## 4. Discussion

To our knowledge this is the first ex vivo study comparing ablated volumes of multi-probe RFA with MWA. In this experiment in bovine livers volumes generated with multi-probe RFA (three electrodes) were significantly larger than volumes generated with a single-probe MWA system. At 4 and 6 min ablation time volumes obtained with the RFA 50 mm setup (three individual CZ) were the largest, while at 8 and 10 min the RFA 20 mm setup (confluent CZ) achieved the largest volumes. The single-probe MWA could not reach the volumes of the RFA setups for any of the ablation times evaluated. This discrepancy is best displayed at the 8 min ablation time, where volumes for the multi-probe RFA setups were larger by 55.4% (confluent CZ) and 39.9% (three individual CZ) compared with single-probe MWA. Similarly, the maximum diameter at 8 min ablation was larger by 16.8% in the RFA setup with confluent CZ compared with the single-probe MWA.

However, the fact that both multi-probe RFA setups were able to match or exceed single-probe MWA when comparing volumes and the confluent CZ RFA setup was able to match or exceed single-probe MWA when comparing diameters is a very important finding, considering that most interventionalists prefer MWA to RFA due to achieving larger ablation volumes/diameters. In fact, it is now undisputed that not only the tumor should be covered by the coagulation zone, but also a sufficient safety margin of 5–10 mm must be obtained [15,16,17,18,19,20,21]. Thus, even for rather small tumors with a 2 cm diameter, coagulation zones should be at least 3 cm, preferably 4 cm in diameter (e.g., in secondary liver malignancies) with correct probe placement in the center of the tumor. These large diameters are almost impossible to achieve using a single RFA electrode (as also demonstrated with the RFA 50 mm setup in this study—see Section 3.3). Implementing the newest MWA probes with very high-power settings (up to 150 W) may be necessary in order to achieve this task in a single-probe setting. For this reason, a recent review [22] highlighting the advantages of single-probe MWA compared to single-probe RFA, concluded that MWA should be the ablative modality of choice for tumors ≥ 3 cm in diameter or in the vicinity of large vessels and that the use of single-electrode RFA should be considered only for a few interventions. However, many institutions now use a variety of different techniques that enable placement of multiple needles for simultaneous or consecutive thermal ablation, overcoming the limitations of single-probe RFA. A major drawback to these multiple needle approaches is certainly the substantially higher complexity and longer overall procedural times compared with conventional single-probe US or CT-guided thermal ablation. In fact, these techniques require sophisticated tools for needle placement such as stereotaxy as in the stereotactic RFA approach developed by the Innsbruck group [4,5]. Furthermore, the risk of puncture-related complications (e.g., bleeding, pneumo-/haemothorax, tumor seeding) is slightly higher [23]. However, multiple smaller coagulation zones allow for a more precise tailoring of the ablation area, particularly for irregular-shaped tumors, and consequently less risk for healthy liver tissue or delicate structures.

Most liver tumors (particularly hepatocellular carcinoma) are spherical. Thus, it is easier to encompass the whole tumor including a sufficient safety margin with a more spherical coagulation zone. In this study, ellipticity indices differed significantly between systems/setups for all evaluated ablation times. MWA and the RFA 20 mm setup (confluent CZ) produced almost round coagulation zones with no significant differences between them in post hoc analysis for any ablation time. Therefore, the advantage of a more spherical coagulation zone using a single-probe MWA may be compensated by confluent coagulation zones with a multi-probe RFA. Coagulation zones of the RFA 50 mm setup on the other hand were more ovoid.

The EI results were very consistent for all ablation systems/setups and had very little to almost no standard deviation. When looking more closely at the results of ablated volumes, we can note very consistent results for MWA with very little to almost no standard deviations. The RFA setups, on the other hand, were more variable with higher standard deviation, particularly for the RFA 50 mm setup at 10 min with a standard deviation of 13.2 mL. The reason for this could be the influence of nearby vessels. With three electrodes, the probability increases that one of the electrodes is placed near a vessel. Furthermore, it is known that MWA is less susceptible to the so-called “heat sink effect” than RFA, in particular monopolar RFA electrodes like the ones used in this experiment [24]. In a clinical setting it is possible to overcome this “heat sink effect” by increased duration and power of ablation, with electrodes being preferentially positioned next to the vessel site and a shorter distance to each other. However, the more accurate prediction of the ablation zone with MWA must be seen as an advantage of this method compared to RFA. 

We are well aware of the limitations of this study. First, all experiments were performed in an ex vivo model in healthy liver tissues. Even though Cassinotto et al. [12] recently disproved the so-called “oven effect”, it is likely that coagulation zones differ depending on tumor heterogeneity (e.g., metastatic vs. primary). Second, this study did not investigate tissue deformation/shrinkage, and earlier studies reported greater tissue deformation with MWA compared with RFA [25,26]. Therefore, the results of this study may be affected by this assumption. Third, even though the experiment was performed in a large basin with circulating saline solution (submersible pump guaranteeing adequate circulation), the impact of the actual in vivo blood circulation, the so-called “heat-sink effect”, cannot be properly assessed—also because of the different velocities of blood circulation in vivo (venous, portal venous, arterial). Fourth, in a clinical setting, such precise, parallel positioning of the electrodes for the confluent coagulation zone with a 20 mm distance is only possible to a limited extent. Fifth, we did not investigate different spacing between electrodes for the confluent coagulation zone RFA setup. However, the choice of the 2 cm distance between electrodes for this setup was based on previous studies with multipolar RFA electrodes [7,27] and our own clinical experience. Although in our experiment all coagulation volumes merged with the 2 cm spacing regardless of the time point, it is not known whether a lower or higher interelectrode spacing would also have resulted in confluent coagulation zones with monopolar RFA electrodes. Therefore, the relationship of distance and time to confluence of coagulation zones for monopolar RFA electrodes should be investigated in a separate experiment.

## 5. Conclusions

In this ex vivo experiment in bovine livers, ablation volumes with the multi-probe RFA system employing three electrodes were significantly larger than those with a single-probe MWA system from the same vendor in both the confluent and three individual coagulation zones configurations. Thus, consistent with the findings of this study it may be reasonable to direct less effort toward the development of new ablation probes with even larger ablation zones and encourage a wider use of multiple needle approaches instead, regardless of the ablation modality. However, further in vivo investigations are required to determine the influence of the heat-sink effect on the resulting ablation volumes.

## Figures and Tables

**Table 1:** Length, Diameter, Volume, and Ellipticity Index for different ablation systems and times; 4 runs at each time point/setup *.

	MWA				RFA 20 mm Confluent CZ				RFA 50 mm * Three Individual CZs			
	4 min	6 min	8 min	10 min	4 min	6 min	8 min	10 min	4 min	6 min	8 min	10 min
Length in mm, mean ± SD	36.5 ± 1.7	39.0 ± 1.2	42.3 ± 2.6	42.5 ± 1.7	40.5 ± 4.2	43.8 ± 2.5	47.8 ± 2.6	48.0 ± 2.5	38.3 ± 3.8	38.2 ± 4.8	39.1 ± 4.0	37.3 ± 4.1
Diameter in mm, mean ± SD	29.0 ± 1.4	36.0 ± 2.8	36.8 ± 2.2	40.5 ± 1.0	33.3 ± 3.9	37.0 ± 1.4	43.0 ± 2.2	43.8 ± 2.5	20.8 ± 2.0	24.5 ± 4.5	25.7 ± 3.8	27.4 ± 5.0
Volume in mL, mean ± SD	16.1 ± 2.2	26.6 ± 4.7	29.8 ± 2.8	36.5 ± 2.6	24.0 ± 7.6	31.5 ± 4.0	46.3 ± 4.9	48.4 ± 7.3	26.5 ± 4.1	38.1 ± 5.8	41.7 ± 4.7	46.0 ± 13.2
Ellipticity Index, mean ± SD	1.3 ± 0.0	1.8 ± 0.1	1.2 ± 0.1	1.0 ± 0.0	1.2 ± 0.1	1.2 ± 0.0	1.1 ± 0.0	1.1 ± 0.0	1.8 ± 0.1	1.6 ± 0.1	1.5 ± 0.1	1.4 ± 0.1

MWA: Microwave ablation; RFA: Radiofrequency ablation; SD: Standard deviation. * *n* = 12 for length, diameter, and volume at the RFA 50 mm setup—see Section 2.5.

## Data Availability

The data that support the findings of this study are available from the corresponding author, YS, upon reasonable request.

## References

[1] Facciorusso A., Di Maso M., Muscatiello N. (2016). Microwave ablation versus radiofrequency ablation for the treatment of hepatocellular carcinoma: A systematic review and meta-analysis. Int. J. Hyperth..

[2] Langenbach M.C. (2019). RFA vs resection of HCC: Exploring the past to improve the future. Eur. Radiol..

[3] Gillams A., Goldberg N., Ahmed M., Bale R., Breen D., Callstrom M., Chen M.H., Choi B.I., de Baere T., Dupuy D. (2015). Thermal ablation of colorectal liver metastases: A position paper by an international panel of ablation experts, the interventional oncology sans frontières meeting 2013. Eur. Radiol..

[4] Laimer G., Schullian P., Bale R. (2021). Stereotactic Thermal Ablation of Liver Tumors: 3D Planning, Multiple Needle Approach, and Intraprocedural Image Fusion Are the Key to Success—A Narrative Review. Biology.

[5] Schullian P., Johnston E.W., Putzer D., Eberle G., Laimer G., Bale R. (2020). Safety and efficacy of stereotactic radiofrequency ablation for very large (≥8 cm) primary and metastatic liver tumors. Sci. Rep..

[6] Haemmerich D., Lee F.T., Schutt D.J., Sampson L.A., Webster J.G., Fine J.P., Mahvi D.M. (2005). Large-Volume Radiofrequency Ablation of ex Vivo Bovine Liver with Multiple Cooled Cluster Electrodes. Radiology.

[7] Clasen S., Schmidt D., Boss A., Dietz K., Kröber S.M., Claussen C.D., Pereira P.L. (2006). Multipolar Radiofrequency Ablation with Internally Cooled Electrodes: Experimental Study in ex Vivo Bovine Liver with Mathematic Modeling. Radiology.

[8] Clasen S., Schmidt D., Dietz K., Boss A., Kröber S.M., Schraml C., Fritz J., Claussen C.D., Pereira P.L. (2007). Bipolar Radiofrequency Ablation Using Internally Cooled Electrodes in Ex Vivo Bovine Liver: Prediction of coagulation volume from applied energy. Investig. Radiol..

[9] Clasen S., Rempp H., Schmidt D., Schraml C., Hoffmann R., Claussen C.D., Pereira P.L. (2012). Multipolar radiofrequency ablation using internally cooled electrodes in ex vivo bovine liver: Correlation between volume of coagulation and amount of applied energy. Eur. J. Radiol..

[10] Rathke H., Hamm B., Güttler F., Rathke J., Rump J., Teichgraber U., de Bucourt M. (2014). Comparison of four radiofrequency ablation systems at two target volumes in an ex vivo bovine liver model. Diagn. Interv. Radiol..

[11] Song K.D., Lee M.W., Park H.J., Cha D.I., Kang T.W., Lee J., Moon J.Y., Rhim H. (2015). Hepatic radiofrequency ablation: *In vivo* and *ex vivo* comparisons of 15-gauge (G) and 17-G internally cooled electrodes. Br. J. Radiol..

[12] Cassinotto C., Denys A., Gay F., Duran R., Hocquelet A., Piron L., Guiu B. (2018). Radiofrequency Ablation of Liver Tumors: No Difference in the Ablation Zone Volume Between Cirrhotic and Healthy Liver. Cardiovasc. Interv. Radiol..

[13] Ruiter S.J.S., Heerink W.J., de Jong K.P. (2019). Liver microwave ablation: A systematic review of various FDA-approved systems. Eur. Radiol..

[14] Mulier S., Ni Y., Frich L., Burdio F., Denys A.L., De Wispelaere J.-F., Dupas B., Habib N., Hoey M., Jansen M.C. (2007). Experimental and Clinical Radiofrequency Ablation: Proposal for Standardized Description of Coagulation Size and Geometry. Ann. Surg. Oncol..

[15] Laimer G., Jaschke N., Schullian P., Putzer D., Eberle G., Solbiati M., Solbiati L., Goldberg S.N., Bale R. (2021). Volumetric assessment of the periablational safety margin after thermal ablation of colorectal liver metastases. Eur. Radiol..

[16] Ahmed M., Solbiati L., Brace C.L., Breen D.J., Callstrom M.R., Charboneau J.W., Chen M.-H., Choi B.I., de Baère T., Dodd G.D. (2014). Image-Guided Tumor Ablation: Standardization of Terminology and Reporting Criteria—A 10-Year Update. J. Vasc. Interv. Radiol..

[17] Lin Y.-M., Paolucci I., O’connor C.S., Anderson B.M., Rigaud B., Fellman B.M., Jones K.A., Brock K.K., Odisio B.C. (2023). Ablative Margins of Colorectal Liver Metastases Using Deformable CT Image Registration and Autosegmentation. Radiology.

[18] Solbiati M., Muglia R., Goldberg S.N., Ierace T., Rotilio A., Passera K.M., Marre I., Solbiati L. (2019). A novel software platform for volumetric assessment of ablation completeness. Int. J. Hyperth..

[19] Kaye E.A., Cornelis F.H., Petre E.N., Tyagi N., Shady W., Shi W., Zhang Z., Solomon S.B., Sofocleous C.T., Durack J.C. (2019). Volumetric 3D assessment of ablation zones after thermal ablation of colorectal liver metastases to improve prediction of local tumor progression. Eur. Radiol..

[20] Shady W., Petre E.N., Do K.G., Gonen M., Yarmohammadi H., Brown K.T., Kemeny N.E., D’Angelica M., Kingham P.T., Solomon S.B. (2018). Percutaneous Microwave versus Radiofrequency Ablation of Colorectal Liver Metastases: Ablation with Clear Margins (A0) Provides the Best Local Tumor Control. J. Vasc. Interv. Radiol..

[21] Wang X., Sofocleous C.T., Erinjeri J.P., Petre E.N., Gonen M., Do K.G., Brown K.T., Covey A.M., Brody L.A., Alago W. (2013). Margin Size is an Independent Predictor of Local Tumor Progression After Ablation of Colon Cancer Liver Metastases. Cardiovasc. Interv. Radiol..

[22] Izzo F., Granata V., Grassi R., Fusco R., Palaia R., Delrio P., Carrafiello G., Azoulay D., Petrillo A., Curley S.A. (2019). Radiofrequency Ablation and Microwave Ablation in Liver Tumors: An Update. Oncologist.

[23] Schullian P., Johnston E., Laimer G., Putzer D., Eberle G., Amann A., Effenberger M., Maglione M., Freund M.C., Loizides A. (2021). Frequency and risk factors for major complications after stereotactic radiofrequency ablation of liver tumors in 1235 ablation sessions: A 15-year experience. Eur. Radiol..

[24] Pillai K., Akhter J., Chua T.C., Shehata M., Alzahrani N., Al-Alem I., Morris D.L. (2015). Heat Sink Effect on Tumor Ablation Characteristics as Observed in Monopolar Radiofrequency, Bipolar Radiofrequency, and Microwave, Using Ex Vivo Calf Liver Model. Medicine.

[25] Amabile C., Farina L., Lopresto V., Pinto R., Cassarino S., Tosoratti N., Goldberg S.N., Cavagnaro M. (2017). Tissue shrinkage in microwave ablation of liver: An ex vivo predictive model. Int. J. Hyperth..

[26] Liu D., Brace C.L. (2019). Evaluation of tissue deformation during radiofrequency and microwave ablation procedures: Influence of output energy delivery. Med. Phys..

[27] Stoffner R., Kremser C., Schullian P., Haidu M., Widmann G., Bale R.J. (2012). Multipolar radiofrequency ablation using 4–6 applicators simultaneously: A study in the ex vivo bovine liver. Eur. J. Radiol..

